# Transformations in river water chemistry following wastewater treatment implementation in a mountain region of the Polish Carpathians

**DOI:** 10.1007/s11356-025-37278-3

**Published:** 2025-12-13

**Authors:** Anna Biernacka, Anna Bojarczuk

**Affiliations:** 1 Institute of Geography and Spatial Management, Faculty of Geography and Geology, Jagiellonian University, Gronostajowa 7, 30-387 Kraków, Poland; 2 Doctoral School of Exact and Natural Sciences, Jagiellonian University, Prof. S. Łojasiewicza 11, 30- 348 Kraków, Poland

**Keywords:** Mountain rivers, Wastewater treatment impact, River water chemistry, Biogenic compounds, Long-term hydrochemical trends, Seasonality

## Abstract

Mountain catchments play a critical role in water supply for lowland regions, contributing significantly to national water resources despite their limited geographic coverage. This study investigates the impact of wastewater treatment plant (WWTP) construction on the hydrochemistry of the Stara River in the Carpathian Foothills (southern Poland). Long-term hydrochemical data (2001–2024) and spatial surveys were analyzed to assess temporal trends and spatial variability before and after the WWTP installation. Results indicate that the WWTP significantly altered river water chemistry, increasing concentrations of conservative ions (Na, Cl) and nutrients (NO_3_, PO_4_), particularly during the vegetative season. Principal Component Analysis (PCA) revealed a shift from diffuse pollution linked to high flow rates prior to 2011, to a dilution-dominated regime after infrastructure development. Seasonal analyses highlighted the winter accumulation of nitrites, likely due to inhibited nitrification under low temperatures. Spatial profiles showed elevated ion concentrations immediately downstream of the WWTP, with partial attenuation further downstream. These findings demonstrate that the implementation of wastewater treatment infrastructure, while improving certain aspects of sanitary safety, may also introduce persistent shifts in river hydrochemistry, particularly under conditions typical for mountain catchments (low buffering capacity, high hydrological variability). To mitigate these effects, we recommend the following: (i) enhanced monitoring of nutrient and ion loads downstream of WWTP outlets, (ii) optimization of treatment processes during low-temperature seasons, and (iii) integration of hydrological modeling into local wastewater management planning. Such measures are essential to maintain ecological resilience and long-term water quality in mountain-fed river systems.

## Introduction

Mountain regions, often referred to as “water towers,” are vital sources of freshwater for adjacent lowlands (Immerzeel et al. 2020). They generate disproportionately high runoff, which is essential for meeting the water demands of downstream areas. Approximately 24% of the world’s lowland population, or about 1.5 billion people, are projected to depend critically on mountain runoff by the mid-twenty-first century. This dependence has increased significantly from 7% in the 1960 s due to rising local water consumption in lowlands (Viviroli et al. 2020). Despite their importance, mountain rivers face challenges such as pollution, climate change, and over-abstraction. Effective management and conservation strategies are necessary to protect these vital water sources and ensure a sustainable water supply for lowland regions.

Mountain areas, despite covering only 3% of Poland’s territory, contribute to 30% of the country’s water resources, primarily through rivers (Niemtur & Pierzgalski 2012). This is significant for water management and supply, especially in lowland regions which are dependent on these water sources. Maintaining good water quality in mountain rivers is of crucial importance, as they serve as a source of clean freshwater for regions located downstream (Wrzesiński 2021). These waters feed river systems that play a significant role in water management, including the supply of drinking water, irrigation of agricultural areas, and industrial operations. Moreover, mountain rivers have a high self-purification capacity and act as a key factor in regulating water quality further downstream, directly affecting river and wetland ecosystems in lowland areas (Pratiwi et al. 2023). Pollution or degradation of mountain watercourses can lead to the accumulation of harmful substances in lowland rivers, deteriorating the living conditions of aquatic organisms and contributing to ecological problems such as eutrophication and changes in ecosystem composition. Therefore, protecting water purity in mountainous areas is a fundamental element of water resource management strategies in Poland.

The quality of surface waters is shaped by a range of natural and anthropogenic factors, among which water and wastewater management plays a key role. The pollution of river waters by municipal and industrial wastewater is one of the major environmental problems worldwide (Morin-Crini et al. 2022; Sarker et al. 2021; Lenart-Boroń et al. 2016a). Effective wastewater treatment reduces the amount of biogenic substances (e.g., nitrogen and phosphorus), limiting eutrophication and the degradation of aquatic ecosystems. This is crucial for the protection of rivers and lakes, which serve as primary sources of drinking water, habitats for aquatic organisms, and essential components of the global water cycle. Untreated or improperly treated wastewater is a source of pathogens and toxic substances that can pose serious threats to human health (Siddharthan et al. 2022; Kesari et al. 2021). In the era of global climate change and increasing water scarcity, efficient water resource management is of paramount importance. Wastewater treatment plants can support a circular economy by enabling the reuse of treated water for agricultural, industrial, or municipal purposes (Hernández-Chover et al. 2023). The construction of sewer systems and wastewater treatment plants is one of the most important measures aimed at reducing water pollution and improving water quality. However, the effectiveness of these solutions depends on various factors, such as the technological advancement of the treatment plant, the wastewater load, and the hydrological conditions of the river (Lenart-Boroń et al. 2019; Bojarczuk et al. 2018). Changes in river water chemistry resulting from the construction of sewage infrastructure and treatment plants can take different directions, depending on the characteristics of the discharged treated wastewater and the self-purification capacity of the river ecosystem (Ledford & Toran 2020). It is therefore essential to understand how the physicochemical parameters of water change along the river’s course and how they fluctuate due to the inflow of treated wastewater. Analyzing these changes along the river allows for an assessment of the actual impact of wastewater treatment plants on water quality and helps identify the key factors shaping their condition. Although the influence of wastewater discharge on river systems has been examined in various hydrological settings, relatively few studies have assessed long-term trends that encompass both the pre- and post-implementation phases of wastewater treatment infrastructure in mountain catchments. Most existing research has focused either on short-term physicochemical variability or on evaluating treatment plant performance without accounting for the cumulative effects on rivers characterized by high hydrological dynamics. By integrating more than two decades of monitoring data with spatial profiling along the river course, this study provides a comprehensive assessment of how wastewater infrastructure influences the hydrochemical regime in a mountain environment. The findings offer new insight into the resilience and recovery potential of mountain-fed rivers subjected to increasing anthropogenic pressure.

The aim of this article is to analyze the impact of wastewater treatment plant construction on trends in river water chemistry and to identify the factors shaping river water quality. Particular attention is given to the spatial variability of the river’s physicochemical parameters before and after the treatment plant, allowing for an assessment of its actual impact on the aquatic environment. The research findings may provide valuable insights for the planning and optimization of wastewater treatment systems in the context of improving surface water quality.

## Methods

### Study area and sampling points

**Fig. 1:**
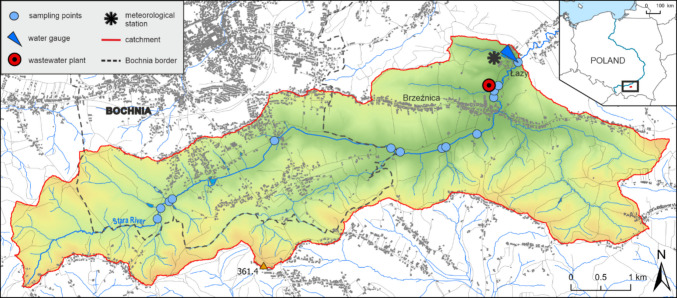
Study area and location of sampling points

The study was conducted in the Stara River catchment (22.3 km^2^), located in the Carpathian Foothills (southern Poland) (Fig. 1). Within the catchment, the two steps of the Carpathian Foothills threshold are clearly visible. The southern part of the catchment consists of resistant Silesian unit flysch, while the northern part comprises flysch and Miocene rocks overlaid by loess-like formations. Consequently, the southern part of the catchment is characterized by steeper slopes and many steep V-shaped valleys forming deep-cutting badlands. In contrast, the northern part of the catchment has gentler slopes and a hilly landscape (Siwek et al. 2017).

The majority of the area (80%) is occupied by Luvisols and Retisols (Albeluvisols) with the presence of a fragipan (Siwek et al. 2021). The remaining 20% is covered by brown, alluvial, and deluvial soils (Skiba et al. 1995). The Stara River catchment is located in a temperate climate zone, within the moderately warm submontane climatic belt (Mirek 2013). The average annual temperature in the study area is 8.9ºC, and the annual precipitation is 720 mm (Siwek et al. 2017).

Almost half of the catchment area, mainly in its southern and western parts, is covered by forests (48%). One-quarter of the area is used for agriculture (24.2%). Approximately 20% is occupied by grasslands, while 8% of the catchment consists of urban areas (BDOT10k). The upper part of the study area is partially located within the administrative boundaries of the city of Bochnia, while the remaining catchment area lies within rural and forested regions. The northwestern areas of the catchment were not connected to the sewer system until 2011. In 2011, a mechanical-biological sewage treatment plant was built, located approximately 700 m upstream of the water gauge station on the Stara River in Łazy.

Long-term hydrochemical monitoring is conducted at the Stara River gauging station, located near the Scientific Research Station of the Institute of Geography and Spatial Management of the Jagiellonian University. Additionally, research was carried out at 13 additional measurement points along the longitudinal profile of the Stara River (Fig. 1).

### Water sample collection and analysis

The study used data on the physicochemical parameters of water and discharge of the Stara River from 2001 to 2024. The period before the construction of the sewage treatment plant covered the years 2001–2010, while the period after the plant’s operation spanned 2011–2023. The analysis of spatial variability in the chemical composition of water along the course of the Stara River was conducted using chemical data from 2022 to 2024. The data on physicochemical parameters included water temperature (Tw), conductivity (EC), pH, mineralization (TDS), total suspended solids (TSS), and the chemical composition of the water.

Field measurements of temperature (Tw), conductivity (EC), and pH were made using portable field meters, and water samples were collected for chemical analysis in 0.5 L polyethylene bottles. The chemical composition of the water was determined using ion chromatography. Using a DIONEX ICS 2000 ion chromatograph and an AS-4 autosampler, major ions (Ca, Mg, Na, K, HCO_3_, SO_4_, and Cl) and nitrogen and phosphorus compounds (NH_4_, NO_3_, NO_2_, and PO_4_) were determined. The water mineralization (TDS) was calculated as the sum of the detected ions. Total inorganic nitrogen (TIN) was also calculated as the sum of N-NH_4_, N-NO_3_, and N-NO_2_.

### Statistical analysis

To determine whether the physicochemical parameters of the water in the Stara River differ significantly before and after the construction of the sewage treatment plant, as well as whether there are significant differences in water chemistry in the river sections above and below the treatment plant, analysis of variance (ANOVA) and the post-hoc Scheffé test were applied at a significance level of *p* = 0.05 (Leščešen et al. 2015). To detect significant trends in the time series of physicochemical parameters of the water in the Stara River before and after the construction of the sewage treatment plant, the Mann–Kendall test (Mann 1945; Kendall 1948) and Sen’s estimator (Sen 1968) were used. These are statistical tools used to analyze long-term changes in hydrological and environmental data. The Mann–Kendall test is a non-parametric statistical method used to assess the direction and significance of trends in time series (Chen et al. 2019). Its advantages include robustness to outliers and no requirement for assumptions regarding the normality of the data distribution. To determine the slope of the trend, Sen’s slope estimator was applied, which allows for estimating the actual magnitude of changes over the study period. It is robust to outliers and does not require the normality of the distributions of the analyzed variables. Principal Component Analysis (PCA) is one of the most commonly used methods for analyzing multidimensional environmental datasets (Chowdhury & Husain 2020). In this study, PCA was used to identify the main factors influencing the Stara River water chemistry before and after the construction of the sewage treatment plant by reducing the number of variables while retaining as much information as possible. The Kaiser criterion was used to select the most important factors, which is one of the most commonly used methods to determine the number of significant principal components in PCA analysis. It is based on the eigenvalue and assumes that only components with eigenvalues greater than 1 should be considered. All statistical analyses were conducted for annual values as well as considering the seasons: the growing season (April–September) and the non-growing season (October–March). The analysis of water chemistry in different seasons allows for a more comprehensive assessment of the dynamics of aquatic ecosystems and the identification of key factors affecting water quality in a temperate climate. Statistical analyses were conducted using Statistica 13 software.

## Results

### Long-term trends and seasonal variations in water chemistry affected by the wastewater treatment plant

**Fig. 2:**
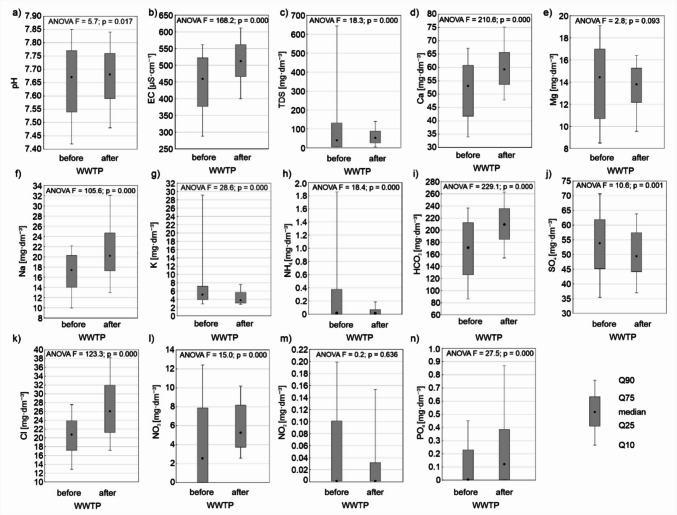
Changes in the physicochemical parameters of the Stara River before and after the construction of WWTP. The graphs show the ANOVA results: *F*-statistic, *p* = significance level

The ANOVA analysis revealed that the construction of the wastewater treatment plant had a significant impact on most physicochemical parameters of Stara River water, except for TSS and Mg. After the construction of the treatment plant, particularly noticeable increases were observed in water parameters such as EC, TDS, Ca, Na, HCO_3_, Cl, NO_3_, and PO_4_ compared to values recorded before the plant’s construction. In contrast, the concentrations of K, NH_4_, SO_4_, and NO_2_ significantly decreased compared to the period without the treatment plant (Fig. 2).

**Fig. 3:**
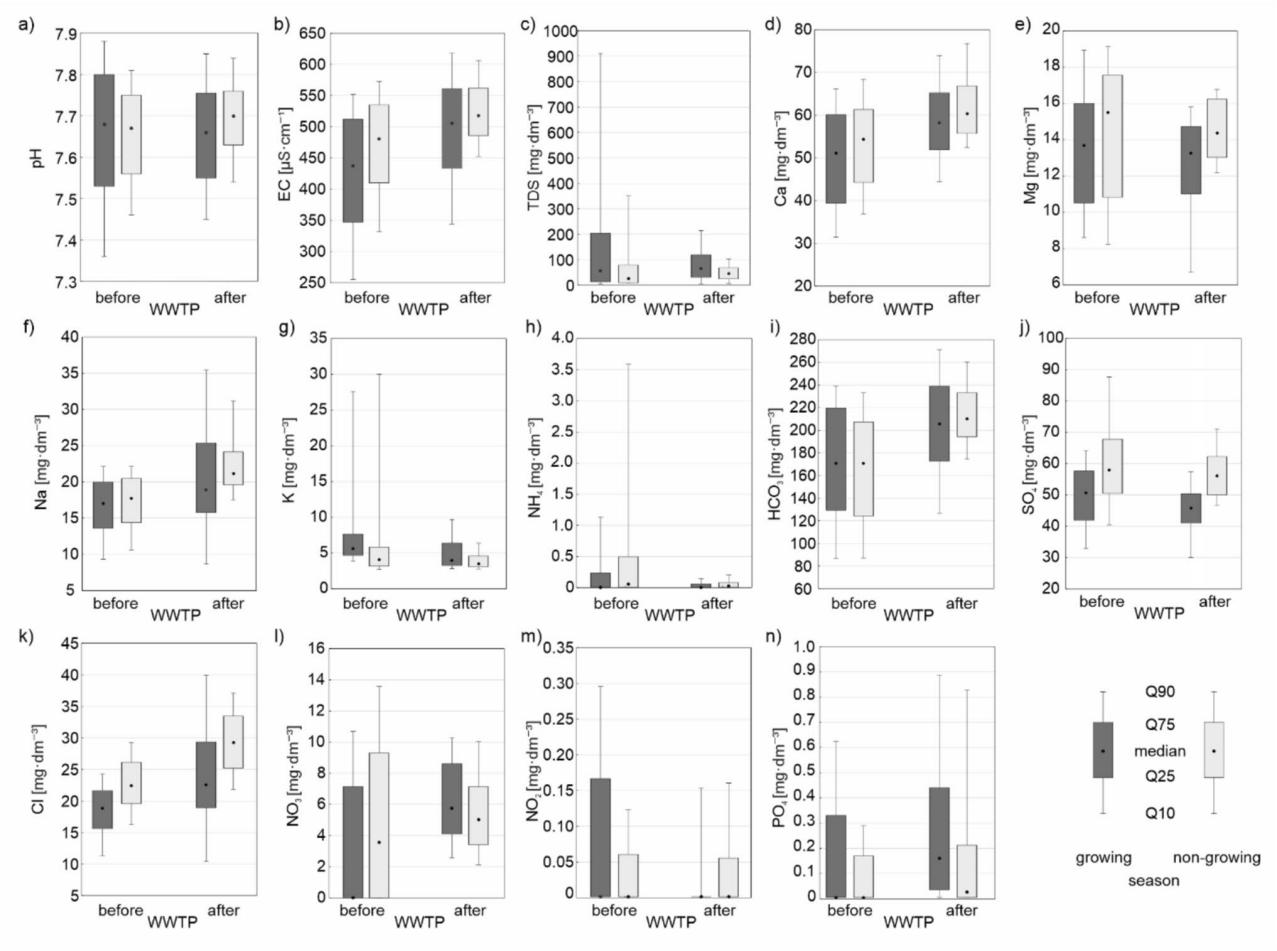
Changes in the physicochemical parameters of the Stara River before and after the construction of the wastewater treatment plant during the vegetation and non-vegetation seasons

The ANOVA analysis conducted for the vegetative and non-vegetative seasons indicated that the construction of the wastewater treatment plant caused more significant differences in Stara River water chemistry during the vegetative season than in the non-vegetative season. During the vegetative season, only pH and TSS showed no significant differences due to the plant’s construction. Conversely, during the non-vegetative season, no significant differences were found between the pre- and post-construction periods for pH, TSS, Mg, SO_4_, and NO_2_. The construction of the wastewater treatment plant significantly increased EC, TDS, and the concentrations of Ca, Na, HCO_3_, Cl, NO_3_, and PO_4_ in water during both vegetative and non-vegetative periods compared to pre-construction values. However, the concentrations of K and NH_4_ in Stara River water significantly decreased (Fig. 3).

**Fig. 4:**
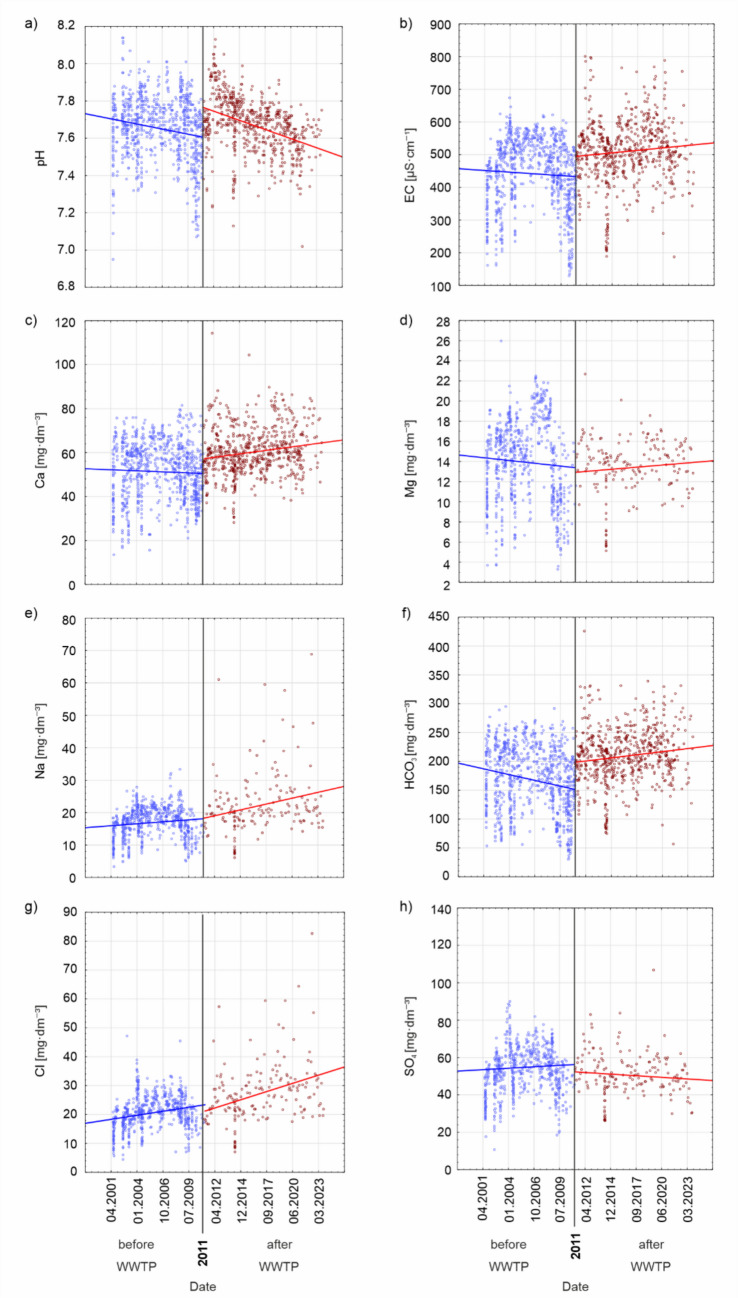
Trends in the physicochemical parameters of the Stara River water before and after the construction of WWTP

The Mann–Kendall test performed on physicochemical parameters before and after the construction of the wastewater treatment plant showed that, on both an annual scale and within the vegetative and non-vegetative seasons, statistically significant trends in the time series of water chemistry were generally absent. However, significant trends were observed almost exclusively for biogenic compounds (Table 1). Before the construction of the treatment plant, significant decreases in NO_2_ and PO_4_ concentrations were observed on an annual scale and during the non-vegetative period. In contrast, during the vegetative season, a significant downward trend was observed only for NO_2_ concentrations. With the wastewater treatment plant in operation, a significant decreasing trend in pH was observed on both annual and seasonal scales in the chemistry of Stara River (Table 1, Fig. 4). Additionally, on an annual scale, a declining trend in NO_3_ concentrations was noted. During the non-vegetative season, a significant decrease in total suspended solids, and SO_4_ concentration, and a significant increasing trend in NO_2_ concentrations were observed.

**Table 1:** Annual and Seasonal Trends in the Physicochemical Characteristics of Stara River Water Before and After the Construction of the Wastewater Treatment Plant

Parameter		pH	EC	TSS	TDS	Ca	Mg	Na	K	NH_4_	HCO_3_	SO_4_	Cl	NO_3_	NO_2_	PO_4_
Non-growing season																
Before WWTP	*S*	3	−5	13	5	−13	−5	−9	−1	−10	−13	1	−9	4	**−25**	**−27**
	*p*	0.858	0.721	0.283	0.721	0.283	0.721	0.474	1.000	0.414	0.283	1.000	0.474	0.785	**0.026**	**0.016**
	Trend	•	•	•	•	•	•	•	•	•	•	•	•	•	↓	↓
	Sen’s slope	0.002	−2.90	8.88	0.65	−0.95	−0.19	−0.33	−0.03	−0.03	−6.21	0.19	−0.46	0.00	**−0.01**	**−0.03**
After WWTP	*S*	**−40**	−16	**−31**	4	20	−20	14	0	20	8	**−38**	32	−20	**36**	−25
	*p*	**0.017**	0.360	**0.020**	0.855	0.246	0.246	0.428	1.000	0.246	0.669	**0.024**	0.059	0.246	**0.027**	0.142
	Trend	↓	•	↓	•	•	•	•	•	•	•	↓	•	•	↑	•
	Sen’s slope	**−0.01**	−1.76	**−2.32**	0.67	0.52	−0.06	0.25	−0.02	0.001	0.44	**−1.30**	0.54	−0.21	**0.01**	−0.02
Growing season																
Before WWTP	*S*	−21	−17	1	5	7	−5	7	11	−20	1	13	13	14	**−28**	−11
	*p*	0.074	0.152	1.000	0.721	0.592	0.721	0.592	0.371	0.085	1.000	0.283	0.283	0.238	**0.014**	0.203
	Trend	•	•	•	•	•	•	•	•	•	•	•	•	•	↓	•
	Sen’s slope	−0.01	5.25	−4.48	5.36	0.34	−0.22	0.21	0.00	−0.02	−2.09	0.83	0.75	0.00	**−0.02**	−0.04
After WWTP	*S*	**−48**	16	−15	22	30	0	18	8	0	22	−22	18	−18	31	−14
	*p*	**0.004**	0.360	0.276	0.200	0.077	1.000	0.300	0.669	1.000	0.200	0.200	0.300	0.300	0.065	0.428
	Trend	↓	•	•	•	•	•	•	•	•	•	•	•	•	•	•
	Sen’s slope	**−0.02**	1.77	−5.87	5.51	0.90	−0.01	0.66	0.16	−0.001	2.97	−0.82	0.88	−0.20	0.01	−0.04
Annual																
Before WWTP	*S*	3	3	−5	5	3	−1	9	−11	−12	−9	5	11	4	**−28**	**−31**
	*p*	0.858	0.858	0.721	0.721	0.858	1.000	0.474	0.371	0.318	0.474	0.721	0.371	0.785	**0.014**	**0.005**
	Trend	•	•	•	•	•	•	•	•	•	•	•	•	•	↓	↓
	Sen’s slope	0.00	4.55	−17.43	2.81	0.40	−0.03	0.18	−0.16	−0.03	−3.98	0.60	0.29	0.00	**−0.02**	**−0.04**
After WWTP	S	**−52**	−2	−15	8	28	−2	18	6	10	10	−28	26	**−34**	27	−22
	p	**0.002**	0.951	0.276	0.669	0.100	0.951	0.300	0.760	0.583	0.583	0.100	0.127	**0.044**	0.112	0.200
	Trend	↓	•	•	•	•	•	•	•	•	•	•	•	↓	•	•
	Sen’s slope	**−0.01**	−0.15	−0.46	2.29	0.64	−0.05	0.61	0.04	0.00	1.33	−0.96	0.89	**−0.24**	0.01	−0.03

*S* Mann–Kendall statistic, *p = *significance level, *↓ *decreasing trend, *↑ *increasing trend, *•* no significant trend; statistically significant trends at *p* = 0.05 are indicated in bold

Despite the lack of statistically significant trends for most major ions in the periods before and after the construction of the wastewater treatment plant (WWTP), clear changes in the direction or slope of the trend lines are visible (Fig. 4). Directional changes are observed for Ca, Mg, and HCO_3_ ions, as their concentrations were decreasing before the construction of the WWTP, whereas after its establishment, their concentrations began to increase.

In the case of SO_4_ ions, the opposite trend is observed—after the construction of the WWTP, their concentrations show a decreasing tendency. The direction of changes in Na and Cl ion concentrations has not changed; however, the slope of the trend line is steeper in the post-construction period, and greater variability in concentrations is observed, especially at higher values.

### Factors shaping water chemistry before and after the construction of the wastewater treatment plant

**Fig. 5:**
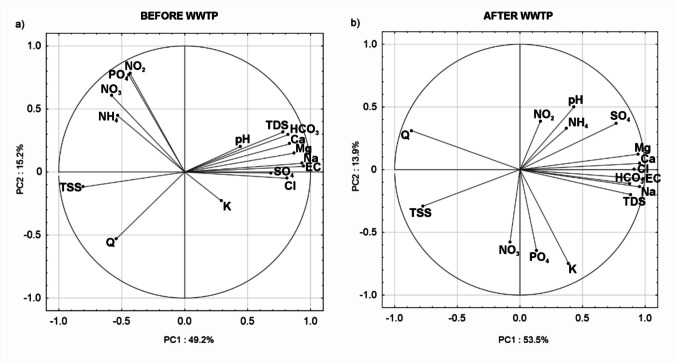
Principal Component Analysis of water chemistry for the periods before and after the construction of the wastewater treatment plant: projection of factor loadings for PC1 × PC2

Based on the Principal Component Analysis (PCA), two main factors shaping the water chemistry of the Stara River were identified for both the periods before and after the construction of the wastewater treatment plant on an annual scale. For the period before the plant’s construction, the identified factors together explain 64.3% of the variability, with the first factor accounting for 49.2% and the second for 15.2% of the variability (Fig. 5). In Factor 1, a negative relationship is observed between TSS, NH_4_, NO_3_, and the flow rate (*Q*) of the Stara River and the concentrations of most major ions, EC, and TDS. It was observed that the higher the river flow, the higher the concentrations of TSS, NH_4_, and NO_3_ in the water, while the concentrations of major ions were lower. In Factor 2, a negative relationship is observed between Q and nitrogen and phosphorus compounds. PCA was also performed separately for the growing and non-growing seasons. In both seasons, two main factors shaping the water chemistry of the Stara River were identified, explaining a similar proportion of the variability: approximately 50% for PC 1 and 16% for PC 2, with relationships consistent with the PCA results on the annual scale.

In the post-WWTP construction period, the total explained variance on an annual scale is slightly higher at 67.4%, with Factor 1 and Factor 2 explaining 53.5% and 13.9% of the variability, respectively (Fig. 5). In PC 1, a negative relationship is observed between TSS, Q, and the concentrations of major ions. In PC 2, a negative relationship is observed between pH and K, NO_3_, and PO_4_. PCA conducted for the growing season after the construction of the treatment plant identified two main factors, explaining 56.5% (PC1) and 14% (PC2) of the variability, respectively. However, for the non-growing season, the analysis revealed three main factors, explaining 42.2% (PC1), 12.2% (PC2), and 10.5% (PC3) of the variability. In the growing season, a negative relationship was observed between *Q*, TSS, and the concentrations of major ions. PC 2 shows a negative relationship between water pH and the concentrations of NO_3_, PO_4_, and K. In the non-growing season, PC 1 indicates a negative relationship between Q and the concentrations of major ions and TSS. In PC 2, the highest factor loadings are observed for NO_3_ and Cl. Finally, PC 3 reveals a negative relationship between PO_4_ and the concentrations of NH_4_, SO_4_, and TSS.

### Impact of the wastewater treatment plant on spatial diversity in water chemistry

**Fig. 6:**
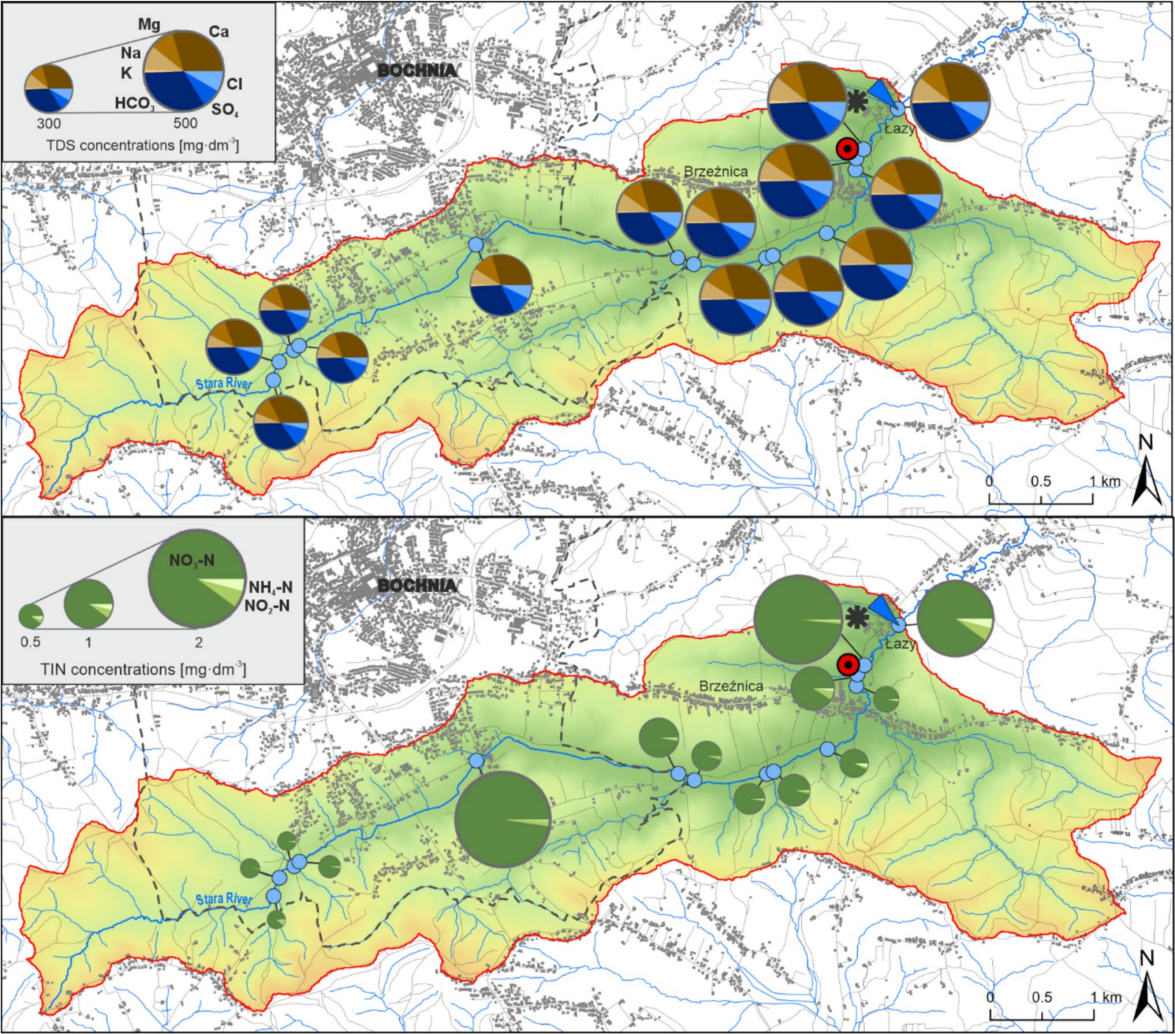
Changes in the structure of chemical composition, concentrations of water mineralization (TDS), nitrogen compounds (NH_4_-N, NO_3_-N, and NO_2_-N), and concentrations of inorganic nitrogen (TIN) along the longitudinal profile of the Stara River

In the Stara River catchment, a gradual increase in water mineralization is observed, from approximately 320 mg·dm^−3^ in the upper reaches to around 500 mg·dm^−3^ in the lower course of the river (Fig. 6). Along the entire river profile, the chemical composition of the water is dominated by HCO_3_ among anions and Ca among cations. Among biogenic compounds, nitrate ions (NO_3_) exhibit the highest concentrations. The influence of effluent from the wastewater treatment plant (WWTP) is manifested in the increased concentrations of all analyzed ions in the Stara River. Among major ions, the most pronounced increase was recorded for Na and Cl, whereas among biogenic compounds, NO_3_ exhibited the greatest rise. Following the WWTP discharge, the relative contributions of Ca, Mg, and HCO_3_ to the ionic structure of river water decreased, while the proportions of other ions increased. The most substantial increase in the share of major ions was recorded for Na and Cl, which rose by 2.8% and 2.1%, respectively. In comparison to the upper course of the Stara River, the contribution of Na and Cl increased by approximately 9% following the inflow from the wastewater treatment plant. Approximately 700 m downstream from the WWTP discharge point, a decrease in the concentration of most ions was observed, indicating dilution and transformation processes. However, ammonium and nitrite concentrations showed an increasing trend along this section of the river, reaching their highest values within the entire longitudinal profile. Notably, their concentrations were even higher than those measured immediately downstream of the WWTP discharge point.

## Discussion

Wastewater treatment plants (WWTPs) constitute one of the primary point sources of pollution in surface waters. Despite their fundamental role in reducing the loads of organic matter, pathogens, and nutrients, effluents from WWTPs often contain substantial quantities of substances that are not fully removed through standard technological processes (Pirsaheb et al. 2014). These include, most prominently: nutrient compounds (nitrogen and phosphorus in both mineral and organic forms), chemical micropollutants (such as pharmaceuticals, personal care products, herbicides, and endocrine-disrupting substances), heavy metals (e.g., Zn, Cu, Pb, and As), and microorganisms, including antibiotic-resistant bacteria (ARB) and antibiotic resistance genes (ARGs) (Jin et al. 2017; Brunsch et al. 2018; Hubeny et al. 2021; Wang J. et al. 2020). The case of the Stara River clearly demonstrates that the construction of a wastewater treatment plant became a significant point source of pollution within the catchment. Following the commissioning of the WWTP, a marked and statistically significant increase in EC and TDS was recorded, along with elevated concentrations of Ca, Na, HCO_3_, Cl, and PO_4_ ions in the Stara River waters. These changes reflect not only the influence of the WWTP itself but also a range of complex biogeochemical and physicochemical processes occurring in the river in response to the inflow of treated wastewater.

The increased concentrations of conservative ions such as Na and Cl, which do not undergo biological transformation, indicate their anthropogenic origin—primarily linked to the presence of detergents, the use of road salt, and infiltration from urbanized areas (Trommetter et al. 2022). The presence of these ions at elevated levels can contribute to the phenomenon known as freshwater salinization syndrome—a chronic salinization of freshwater bodies that results in alterations to trophic structure and the functioning of entire aquatic ecosystems (Maas et al. 2023). It is also important to note that chloride ions are particularly difficult to remove during wastewater treatment because they are not available for microbial degradation and represent the most chemically stable form of chlorine in aqueous systems. As a result, standard physicochemical treatment techniques often fail to reduce Cl⁻ concentrations to environmentally safe levels, and more advanced methods such as enhanced precipitation or advanced oxidation processes are required to achieve effective removal. This highlights why Cl⁻ enrichment in surface waters persists even in catchments equipped with modern wastewater treatment infrastructure (Dou et al. 2022).

In contrast, the observed increase in concentrations of biogenic compounds—particularly NO_3_ and PO_4_—indicates the effectiveness of nitrification processes within the treatment plant, while simultaneously suggesting limited retention of nutrients within the river itself. Aissa-Grouz et al. (2015) noted that the transformations of NH_4_ → NO_2_ → NO_3_ under oxic conditions favor the presence of more mobile and persistent nitrogen forms in aquatic environments. On the other hand, the lack of active denitrification and the low sorption capacity of riverbed sediments can result in secondary eutrophication (Zhou et al. 2022). The declining trends in concentrations of NH_4_, NO_2_, and SO_4_ in the Stara River may be attributed both to their efficient removal at the treatment plant and to subsequent in-stream transformations—such as further nitrification (in the case of NH_4_ and NO_2_) or sulfate reduction to hydrogen sulfide (H_2_S) under low redox potential conditions (Deng et al. 2018). Such reductive processes are often observed in zones of flow stagnation and in bottom sediments enriched in organic matter. Moreover, the substantial decline in potassium concentrations suggests its intensive biological uptake by aquatic microorganisms, mainly autotrophs and heterotrophs associated with biofilms and periphyton communities. As noted by Wang H. et al. (2020), potassium—due to its limited sorption and weak mineral association—can serve as a sensitive indicator of biological activity in freshwater environments. Taken together, the observed chemical changes in the Stara River suggest that oxidative processes dominate in the watercourse following the inflow of treated wastewater. These processes lead to the transformation of reduced nitrogen and sulfur species into their oxidized forms, which are more stable and more difficult to remove from the aquatic ecosystem. According to Li et al. (2021), such chemical transformation exerts a direct influence on the aquatic microbiome by promoting its structural homogenization, reducing the abundance of sensitive species, and disrupting the trophic balance of the river ecosystem as a whole.

In the analysis of seasonal effects on the functioning of wastewater treatment plants (WWTPs) and the shaping of surface water chemistry, the variable climatic conditions characteristic of the temperate zone play a crucial role. Particularly pronounced differences are observed between the growing season (April–September) and the non-growing (winter) season (October–March), which are reflected in both the intensity of biogeochemical processes and the operational efficiency of WWTPs. Research conducted in the Stara River catchment demonstrated that selective accumulation of NO_2_ occurs during the colder months, while this phenomenon is not observed during the summer period. This mechanism can be explained by the seasonal imbalance in the activity of microorganisms involved in nitrification processes. At low water temperatures (< 10 °C), typical of winter months, the activity of nitrite-oxidizing bacteria from the genera Nitrobacter and Nitrospira—responsible for the conversion of NO_2_ to NO_3_—is significantly reduced. Meanwhile, the first stage of nitrification—transformation of NH_4_ to NO_2_ by Nitrosomonas bacteria—remains active, leading to the transient accumulation of NO_2_ in the water column (Zajac et al. 2023). Comparable seasonal effects have also been observed in systems based on facultative ponds, where algal photosynthetic activity and associated bacterial processes decline markedly during colder months (Almasi et al. 2015). As demonstrated in a year-long survey of primary and secondary facultative ponds, effluent algal BOD ratios were significantly lower in winter than in summer, indicating reduced biological treatment efficiency under low temperatures and limited light conditions. These findings further support the interpretation that seasonal microbial dynamics strongly influence the composition of treated wastewater entering river systems. Reduced biological activity in the stream during winter, limited uptake of nutrients by autotrophs, and the absence of intensive photosynthesis further amplify this effect. At the same time, higher oxygen solubility in cold water maintains favorable conditions for oxidation processes; however, in the absence of a complete microbiological nitrogen transformation cycle, intermediate forms accumulate. In summer, the situation changes—rising temperatures stimulate the activity of Nitrobacter, resulting in efficient conversion of NO_2_ to NO_3_, while increased phytoplankton and benthic biofilm activity effectively reduces nitrogen compound concentrations in the water (Comer-Warner et al. 2020). Similar phenomena have been reported in studies from other climatic regions. For instance, Schliemann et al. (2021), in their analysis of the South Platte River in Colorado, demonstrated that in winter—when treated wastewater accounted for up to 90% of total river flow—NO_2_ concentrations rose sharply, while in summer, levels remained below detection limits. Similarly, Romero et al. (2013) emphasized that it is the seasonal dynamics of microbial processes, rather than flow variability alone, that determine the spatial and temporal extent of WWTP’s influence on nitrogen cycling in riverine ecosystems.

The analysis of wastewater discharges from treatment plants also reveals significant differences depending on the nature of the inflow—continuous versus intermittent. In the case of a continuous and steady wastewater inflow into the river, the system tends to reach a quasi-stable state. Under such conditions, spatially heterogeneous but predictable zones of biogenic compound concentrations develop, while self-purification processes—including nitrification, denitrification, sorption, and biological ion uptake—operate under conditions of relative equilibrium (Rueda et al. 2002). In such scenarios, oxidized nitrogen forms such as NO_3_ dominate, whereas the concentrations of NH_4_ and NO_2_ remain moderate and stable. In contrast, rivers receiving intermittent discharges—arising from diurnal or weekly fluctuations in WWTP operation, hydraulic overloads, or seasonal tourist peaks—experience more dynamic and unstable conditions. In such systems, substantial fluctuations in NH_4_ and NO_2_ concentrations are frequently observed, often manifesting as episodic peaks that indicate disruptions in the nitrification cycle. As noted by Lenart-Boroń et al. (2016b), during periods of high tourist pressure, rivers such as the Białka may experience extreme diurnal increases in NH_4_ concentrations—up to 35-fold within a single day. These events disrupt the physicochemical equilibrium of the stream, reduce buffering capacity, and destabilize the microbiological structure. Under such dynamically changing inflow conditions from WWTPs, the activity of denitrifying bacteria and the composition of the biofilm microbiome may fluctuate, while the spatial extent of WWTP influence becomes less predictable and exhibits significantly higher variability. Rueda et al. (2002) demonstrated that such phenomena can lead to the temporary dominance of opportunistic bacteria, including pathogenic and antibiotic-resistant strains, which significantly impair the resilience of riverine ecosystems to subsequent environmental stressors.

The impact of wastewater discharges from treatment plants on river water chemistry along the longitudinal profile of a stream exhibits marked spatial variability. Changes in ion concentrations and micropollutants are not confined solely to the immediate vicinity of the discharge point, but can influence water quality over considerable distances downstream—depending on a range of environmental and technological factors. Key determinants of the spatial extent of WWTP influence include the technological characteristics of the facility (e.g., treatment efficiency, types of purification processes, and effluent quality), the discharge volume relative to river flow, as well as channel morphology, flow velocity, water retention, and the presence of marginal zones such as riparian vegetation. Climatic factors—such as rainfall intensity, drought frequency, and the amplitude of seasonal flow variability—also play an important role (Comber et al. 2020; Castelar et al. 2022).

In the case of the Stara River, spatial variation in WWTP influence was also observed. Immediately downstream of the discharge point, the highest concentrations of conservative ions such as Na and Cl, as well as biogenic compounds (NO_3_, PO_4_), were recorded. The relative proportions of ions such as Ca, Mg, and HCO_3_ declined, suggesting a dilution effect of natural hydrochemical constituents by the influx of wastewater with a distinct ionic composition. These changes were particularly evident in the percentage composition analysis—for instance, the proportion of Na increased by over 2.8% and Cl by 2.1%.

Processes occurring further downstream from the WWTP discharge include both dilution and biological transformations—such as nitrification, denitrification, sorption, and sedimentation. The intensity of these processes depends on the physical characteristics of the riverbed, the presence of marginal zones and riparian vegetation, as well as prevailing hydrological conditions (Xia et al. 2018). During low-flow periods, processes such as retention and local uptake of compounds by biofilms may dominate, whereas during high-flow conditions, the transport of substances over longer distances downstream becomes more significant (Bernal et al. 2020; Lupon et al. 2016).

It is noteworthy that the influence of wastewater treatment plants (WWTPs) on river water composition extends beyond physicochemical variables. It also encompasses a wide array of ecological and microbiological processes, highlighting the need for integrated chemical, biological, and molecular analyses in studies addressing WWTP discharge impacts. Changes in the microbiome, particularly in biofilms and benthic sediments, may persist over the long term and exhibit greater resistance to self-purification processes than chemical parameters (Del Olmo et al. 2025). Research conducted across different global regions documents significant variation in the spatial extent of treated effluent impact. Ribot et al. (2012), examining a river in Catalonia, demonstrated that NH_4_ concentrations rose sharply following WWTP discharge, but significantly declined within just 150 m—indicating a strong short-range self-purification effect. In contrast, Aristi et al. (2015) reported that despite dilution and biological transformations, elevated concentrations of EC, NH_4_, PO_4_, dissolved organic carbon, and pharmaceuticals persisted even 4 km downstream from the discharge point. In studies conducted on the Onyar River, Del Olmo et al. (2025) found that concentrations of micropollutants such as pharmaceuticals, heavy metals, and microplastics exhibited significant differences even 2.8 km downstream. Importantly, only after approximately 525 m was partial recovery observed in the composition of the biofilm microbiome and the presence of antibiotic resistance genes (ARGs). This phenomenon suggests that the microbiological structure of the riverine environment exhibits a greater temporal inertia than the chemical composition of the water itself. Seasonal dynamics further modulate the spatial extent of WWTP influence. Higher decay rates of microbial indicators during the dry season shorten self-depuration distances (SDDs), increasing the river’s natural purification capacity. In contrast, during wet periods, lower decay rates result in longer SDDs; for example, the distance required for the attenuation of sulfite-reducing clostridia spores can be as short as 3 km in dry conditions, while the human-specific Bifidobacterium marker may persist for up to 15 km during high-flow wet season events. These patterns indicate that hydrological regime and microbial decay kinetics jointly govern the effective spatial footprint of wastewater discharge in rivers (Pascual-Benito et al. 2020). Further, in research on the Enoree River (USA), Andersen et al. (2014) showed that a WWTP located in the upper section of the river significantly increased salinity and the concentrations of most ions (including Cl, Na, and SO_4_), with its effects detectable as far as the lower reaches—over a distance exceeding 135 km. In contrast, WWTPs situated downstream in the river system had minimal influence on the overall chemical composition of the water. The authors emphasized that dilution was the principal mechanism driving the reduction in dissolved substance concentrations; however, sorption processes in sediments and biological uptake of nitrates and phosphates by autotrophs also played a significant role.

Principal Component Analysis (PCA) conducted for the period preceding the construction of the wastewater treatment plant (WWTP) in the Stara River catchment revealed that the dominant factor (PC1) shaping river water chemistry was the intensification of pollutant inflows associated with increased river discharge. Higher flow rates were positively correlated with elevated concentrations of total suspended solids (TSS) and biogenic compounds—particularly nitrogen forms (NH_4_, NO_3_) and phosphorus. These findings clearly indicate the significant influence of diffuse pollution sources during the analyzed period, attributable to the lack of sewage infrastructure and widespread use of leaking household septic tanks. During storm events, these tanks frequently overflowed, resulting in the direct release of untreated wastewater into surface waters (Suchowska-Kisielewicz & Nowogoński 2021; Richards et al. 2016). Within the same principal component, negative correlations were observed between flow and concentrations of major ions such as Ca, Na, and HCO_3_, suggesting the dominance of dilution processes during high-flow conditions. This pattern is typical of mountain rivers, where flood events are often short-lived but intense, and the prevailing mechanism involves the flushing of biogenic materials and suspended solids from surface sources, alongside dilution of the river’s natural mineral background. Following the commissioning of the WWTP in 2011 and the connection of most households to the sanitary sewer network, the structure of factors influencing river water chemistry changed significantly. In the PCA analysis for the post-2011 period, the previous positive correlation between discharge and elevated concentrations of biogenic compounds was no longer observed. On the contrary, a clear dilution pattern was detected, wherein increased flow was associated with decreasing concentrations of major ions, without any corresponding rise in NO_3_ or PO_4_ levels. This pattern, typical of systems served by well-functioning sewer infrastructure, indicates that river water was no longer directly impacted by pollutant loads from diffuse sources, reflecting the effectiveness of infrastructural interventions within the catchment. Additionally, reductions in biogenic concentrations during high-flow conditions may also have been strengthened by changes in land use within the Stara River basin. Recent research by Bojarczuk and Biernacka (2025) documented a decline in agricultural land cover in the catchment, which likely contributed to reduced runoff-driven inputs of sulfates and nitrogen compounds from arable fields, further supporting the observed decrease in nutrient loads during flood events.

The transition from a heavily anthropogenically impacted system to a more chemically stable riverine ecosystem was also supported by the reduced seasonal variability and diminished diurnal fluctuations in ammonium (NH_4_) concentrations, which prior to modernization had shown high amplitude. The impact of sanitary infrastructure development in the Stara River catchment is also consistent with global trends documented in the literature. As noted by Wang Q. et al. (2020), newly constructed or upgraded WWTPs demonstrate a significant capacity to reduce concentrations of total organic nitrogen and to decrease the abundance of nitrifying bacteria. Simultaneously, an increase is often observed in the proportion of denitrifying bacteria and microorganisms employing dissimilatory nitrate reduction to ammonium (DNRA) pathways, which can enhance overall river water quality by accelerating in-stream biogeochemical transformations.

However, not all municipal infrastructure upgrades yield the anticipated outcomes. Studies on the Białka River (Lenart-Boroń et al. 2019) showed that despite the modernization of the WWTP and reductions in TDS and NH_4_ concentrations, phosphorus levels remained extremely high. Moreover, the upgrade did not improve the microbiological quality of the treated effluent, which was attributed to increasing WWTP load due to tourism growth in the Podhale region and heightened demographic pressure.

The above examples underscore the importance of a comprehensive approach when evaluating WWTP performance—one that incorporates not only physicochemical and microbiological parameters but also the broader socio-economic context. Under conditions of hydraulic or organic overloading, even modernized WWTPs may exhibit reduced treatment efficiency, potentially leading to renewed degradation of the receiving water bodies’ quality.

## Conclusion

This study highlights the significant and multifaceted impact of wastewater treatment plants (WWTPs) on riverine ecosystems in mountain catchments, which are critical sources of freshwater for downstream regions. The case of the Stara River illustrates that the construction and operation of a WWTP can substantially alter water chemistry, particularly by increasing concentrations of conservative ions (e.g., Na and Cl) and nutrients (e.g., NO_3_ and PO_4_). These changes are not limited to the immediate vicinity of the discharge point but extend downstream, with variability influenced by seasonal conditions, hydrological regimes, and the nature of wastewater inflow.

Principal Component Analysis revealed a shift from a pollution regime dominated by diffuse sources prior to WWTP installation to a more stable, dilution-driven system thereafter. Seasonal analyses further demonstrated that winter conditions hinder complete nitrification, leading to transient nitrite accumulation. Moreover, spatial profiles confirmed both physicochemical and microbiological responses to effluent inputs, emphasizing the importance of integrating molecular and ecological assessments in water quality monitoring.

Although modern WWTPs improve water quality under optimal conditions, their effectiveness can be compromised by hydraulic overload or inadequate nutrient removal. Thus, comprehensive, context-sensitive management strategies are essential for maximizing the ecological benefits of wastewater infrastructure, particularly in sensitive headwater catchments that support downstream water security.

## Data Availability

The datasets generated and analyzed during the current study are available from the corresponding author upon request.
